# Sex- and age-specific impacts of smoking, overweight/obesity, hypertension, and diabetes mellitus in the development of disabling dementia in a Japanese population

**DOI:** 10.1265/ehpm.22-00187

**Published:** 2023-02-03

**Authors:** Mari Tanaka, Hironori Imano, Mina Hayama-Terada, Isao Muraki, Kokoro Shirai, Kazumasa Yamagishi, Takeo Okada, Masahiko Kiyama, Akihiko Kitamura, Yoshihiro Takayama, Hiroyasu Iso

**Affiliations:** 1 Department of Social Medicine, Osaka University Graduate School of Medicine, Osaka, Japan; 2 Department of Public Health, Kindai University Faculty of Medicine, Osaka, Japan; 3 Osaka Center for Cancer and Cardiovascular Disease Prevention, Osaka, Japan; 4 Yao City Public Health Center, Yao City Office, Osaka, Japan; 5 Institute of Global Health Policy Research (iGHP), Bureau of International Health Cooperation, National Center for Global Health and Medicine, Tokyo, Japan; 6 Department of Public Health Medicine, Faculty of Medicine, and Health Services Research and Development Center, University of Tsukuba, Ibaraki, Japan

**Keywords:** Dementia, Cardiovascular risk factors, Hazard ratio, Population attributable fractions

## Abstract

**Background:**

Sex- and age-specific impacts of cardiovascular risk factors on the development of dementia have not been well evaluated. We investigated these impacts of smoking, overweight/obesity, hypertension, and diabetes mellitus on the risk of disabling dementia.

**Methods:**

The study participants were 25,029 (10,134 men and 14,895 women) Japanese aged 40–74 years without disabling dementia at baseline (2008–2013). They were assessed on smoking status (non-current or current), overweight/obesity (body mass index ≥25 kg/m^2^ and ≥30 kg/m^2^, respectively), hypertension (systolic blood pressure ≥140 mmHg, diastolic blood pressure ≥90 mmHg or any antihypertensive medication use), and diabetes mellitus (a fasting serum glucose ≥126 mg/dL, non-fasting glucose ≥200 mg/dL, hemoglobin A1c ≥6.5% by the National Glycohemoglobin Standardization Program or glucose-lowering medication use) at baseline. Disabling dementia was identified as the level of care required ≥1 and cognitive disability grade ≥IIa according to the National Long-term Care Insurance Database. We used a Cox proportional regression model to estimate hazard ratios and 95% confidence intervals (95% CIs) of disabling dementia according to the cardiovascular risk factors and calculated the population attributable fractions (PAFs).

**Results:**

During a median follow-up of 9.1 years, 1,322 (606 men and 716 women) developed disabling dementia. Current smoking and hypertension were associated with a higher risk of disabling dementia in both sexes, whereas overweight or obesity was not associated with the risk in either sex. Diabetes mellitus was associated with a higher risk only in women (p for sex interaction = 0.04). The significant PAFs were 13% for smoking and 14% for hypertension in men and 3% for smoking, 12% for hypertension, and 5% for diabetes mellitus in women. The total PAFs of the significant risk factors were 28% in men and 20% in women. When stratified by age, hypertension in midlife (40–64 years) was associated with the increased risk in men, while diabetes mellitus in later-life (65–74 years) was so in women.

**Conclusions:**

A substantial burden of disabling dementia was attributable to smoking, and hypertension in both sexes and diabetes mellitus in women, which may require the management of these cardiovascular risk factors to prevent dementia.

## Introduction

Dementia has emerged as a major public health issue worldwide [1]. In Japan, a super-aging society, 15% of older people aged 65 and over had dementia in 2012, and the prevalence is estimated to be more than double by 2060 [2]; thus, the prevention of dementia is urgently needed.

The 2020 Lancet Commission reported that approximately 40% of worldwide dementia could be prevented by 12 risk factors identified as having consistent evidence for the association from meta-analytic reviews: less education for early life (<45 years), hearing loss, traumatic brain injury, hypertension, alcohol consumption (≥21 drinks/week), obesity (body mass index [BMI] ≥30 kg/m^2^) for middle life (45–65 years), smoking, depression, social isolation, air pollution, physical inactivity, and diabetes mellitus for late life (≥65 years) [3].

Of the above 12 risk factors, smoking, obesity, hypertension, and diabetes mellitus are mandated for annual screening in the Japanese health system [4], and are well-known risk factors for cardiovascular disease [5]. Cardiovascular disease often occurs before dementia, including vascular dementia as well as Alzheimer’s disease [6, 7]. Vascular dementia accounts for a higher percentage in Japan than in Western countries [8], provably due to the high burden of stroke in Japan. Thus, preventing and controlling for cardiovascular risk factors could reduce risk of dementia. However, only one study of 8,563 older Japanese aged ≥65 years estimated the burden of dementia attributable to these risk factors via the population attributable fraction (PAFs) [9]; approximately one-third of disabling dementia cases were attributed to seven major risk factors (smoking, physical inactivity, hypertension, diabetes mellitus, obesity with BMI of ≥30 kg/m^2^, severe psychological distress, and low educational attainment).

That study, as well as the 2020 Lancet Commission, did not provide the sex-specific impacts of cardiovascular risk factors on dementia. The prevalence and incidence of vascular dementia was lower in women than in men [10, 11], while that of Alzheimer’s disease was reported to be higher in women than in men probably because women live longer than men [12]. Women with a history of cardiovascular diseases are more likely to develop Alzheimer’s disease, but not vascular dementia compared with men [13]. The sex difference in the prevalence and incidence of dementia subtypes suggests that some risk factors associated with risk of dementia may differ between men and women. Raised blood pressures and diabetes mellitus were associated stronger with risk of dementia in women than in men [14, 15]. On the other hand, smoking rate, higher blood pressure levels, and higher prevalence of diabetes mellitus were observed in men than in women [16, 17]. Especially, smoking rate was much higher in Japanese men than in Japanese women [17]. These findings warrant further studies to evaluate the sex-specific burden of dementia attributable to cardiovascular risk factors.

In addition, the incidence of dementia increases with age while levels of cardiovascular risk factors vary with age. The Framingham Heart Study reported that systolic blood pressure level and diabetes mellitus at age 55, diabetes mellitus at ages 70, 75, and 80 were regarded as vascular risk factors for dementia [18]. Therefore, it is necessary to evaluate the sex- and age-specific risk factors and their impacts and attributable to dementia.

Our prior hypothesis is that cardiovascular risk factors and their impacts on the development of dementia vary by sex and age.

In the present prospective cohort study, we examined the sex- and age-specific impacts of smoking, overweight/obesity, hypertension, and diabetes mellitus on the risk of dementia in a Japanese population.

## Methods

### Design, setting, and participants

We used the data from the collaborative research between Yao City, Osaka Prefecture, and Osaka University, which aimed to promote health among Yao City residents. The population size of Yao City, Osaka Prefecture (southwestern Japan), was 266,143 citizens (128,974 men and 137,169 women) on 31 March 2008. The percentage for ages 40–74 years accounted for 46% of the total census population, which was similar to the percentage in the 2010 national census population. Approximately 10% of people aged 40–74 years (n = 12,122; 4,824 men and 7,298 women) received health check-ups under the national health insurance in fiscal 2008.

**Fig. 1:**
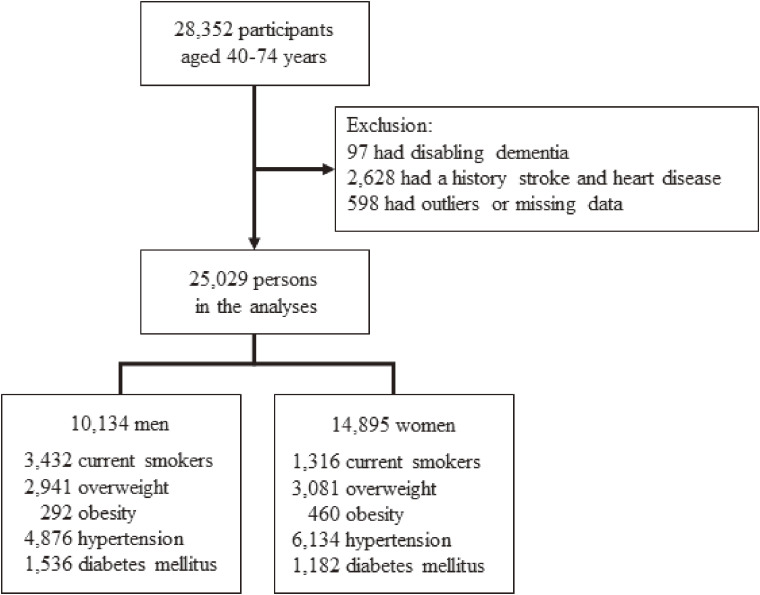
Flow chart for selection of the study participants.

We used the data from the annual national health check-up, the national long-term care insurance, and notification of moving-out, moving-in, and death in this collaborative research. The present study was a prospective cohort study of individuals aged 40–74 years who underwent health check-ups under the national health insurance between April 2008 and March 2013. There were initially 28,352 participants at baseline. Individuals were excluded if they had disabling dementia (n = 97) or had a history of cardiovascular disease, including stroke and heart disease from a self-reported questionnaire (n = 2,628), or had outliers in the anthropometric measurements or laboratory tests or missing data (n = 598). Finally, 25,029 participants (10,134 men and 14,895 women) were enrolled in the present study (Fig. 1). The protocol was approved by the ethics committees of Osaka University, Yao City, and Osaka Center for Cancer and Cardiovascular Disease Prevention.

### Definition of disabling dementia and follow-up

We used the data from the screening judgment results for long-term care requirement certification under the national long-term care insurance system established in 2000 [19, 20].

The certification of the long-term care requirement was provided based on the evaluation results of the applicants’ activities of daily living, psychophysical condition, and cognitive function from a national standardized in-home assessment by trained investigators in the municipality and a medical assessment by primary care physicians. In the certification, a person’s level of required care was classified as preventive support level of 1 or 2, long-term care levels of 1 to 5, and a person’s cognitive disability grade was classified as non-cognitive symptoms, I, IIa, IIb, IIIa, IIIb, IV, and M (M = needs treatments in a specialized medical facility because of serious cognitive symptoms) [19, 20]. Disabling dementia was defined as the level of long-term care required ≥1 and cognitive disability grade ≥IIa in the present study as defined in the previous study [21]. The validation of the criteria previously confirmed that the sensitivity and specificity values were 73% and 96%, respectively, compared with the diagnosis by neuropsychiatrists [22].

The follow-up lasted until the end of December 2018 and was terminated at the first incident of disabling dementia, exit from the community, or death.

### Risk factors and baseline examination

In Japan, screening for smoking, being overweight, hypertension, diabetes mellitus, and dyslipidemia have been mandated in annual health check-ups for persons aged 40–74 years since 2008 to prevent cardiovascular disease [4]. Of these risk factors, we focused on smoking, obesity (BMI ≥30 kg/m^2^), hypertension, and diabetes mellitus, which were reported as the risk factors with the most consistent evidence on the association of dementia in the 2020 Lancet Commission [3]. The present study also examined the association between overweight (BMI ≥25 kg/m^2^) and the risk of disabling dementia because the prevalence of obesity in Japan is very low compared to that in Western countries [23].

The dataset in the present study includes information on general characteristics (sex, date of birth, and date of health check-up), a self-administered questionnaire on lifestyle (smoking status and alcohol consumption), anthropometric measurements (height, weight, and BMI), blood pressure (systolic blood pressure [SBP], diastolic blood pressure [DBP]), laboratory values (fasting serum glucose, non-fasting serum glucose, hemoglobin A1c [HbA1c], low-density lipoprotein cholesterol [LDL-C], high-density lipoprotein cholesterol [HDL-C], and protein in the urine), medication use (antihypertensive medication, glucose-lowering medication, and lipid-lowering medication), and medical history (stroke, heart disease, and chronic kidney disease including artificial dialysis). The baseline HbA1c value was determined using the previous Japanese standard substance and measurement (Japan Diabetes Society [JDS]). Therefore, the HbA1c value was estimated as the National Glycohemoglobin Standardization Program (NGSP) equivalent value calculated using the following formula: HbA1c (NGSP) (%) = 1.02 × HbA1c (JDS) (%) + 0.25% [24].

Overweight and obesity were defined as BMI of ≥25 kg/m^2^ and ≥30 kg/m^2^, respectively [25]. Hypertension was defined as SBP ≥140 mmHg, DBP ≥90 mmHg or any antihypertensive medication use [26]. Diabetes mellitus was defined as a fasting serum glucose ≥126 mg/dL (≥7.0 mmol/L) or non-fasting glucose ≥200 mg/dL (≥11.1 mmol/L), HbA1c (NGSP) ≥6.5% or glucose-lowering medication use [27].

### Statistical analysis

The values of baseline characteristics according to disabling dementia during follow-up in men and women were reported as means (standard deviations) for continuous variables and percentages for categorical variables. The analysis of covariance after adjustment for age (continuous) was used to compare the presence or absence of disabling dementia. Person-years were calculated as the sum of individual follow-up time until the occurrence of disabling dementia, death, exit from the community, or the end of follow-up, whichever occurred first.

Cox proportional hazards regression models were used to estimate the sex- and age-specific hazard ratios (HRs) and 95% confidence intervals (CIs) for the risk of incident disabling dementia according to risk factors (smoking, overweight/obesity, hypertension, and diabetes mellitus). We divided the participants into two groups (40–64 and 65–74 years) based on the median age (65 years) of the participants. To estimate the sex-specific HRs, the initial model was adjusted only for age, while the multivariable model was adjusted for age, smoking status (non-current or current), drinking status (non-current or current), obesity (yes or no), hypertension (yes or no), diabetes mellitus (yes or no), LDL-C, HDL-C, and lipid-lowering medication use (yes or no). The test for effect modification by sex in the analysis of each risk factor was conducted with interaction terms generated by multiplying the categorical variable of each risk factor (0 or 1) by sex (0 or 1). In the multivariable analysis, the test for effect modification by age were performed with interaction terms generated by multiplying the categorical variable of each risk factor (0 or 1) by age group (0 or 1).

We estimated the sex- and age-specific multivariable-adjusted PAFs (95% CIs) of incident disabling dementia according to each risk factor, which is the proportion of disabling dementia events in the population that would be attributable to a particular risk factor. We used a category-specific attributable fraction with the formula pd*i* (1 − 1/RR*i*), which produces internally valid estimates when confounding exists [28], where pd*i* represents the proportion of total events in the population arising from the *i*th exposure category and RR*i* is the multivariable-adjusted HR for the *i*th exposure category relative to the unexposed group. We also calculated approximate estimates of the 95% CI for the PAF [29]. The sum of the category-specific attributable fractions was calculated using the following formula [28]:

$$\sum_{i}pdi\left(1-\frac{1}{RRi}\right)$$

All analyses were conducted using SAS version 9.4 (SAS Institute, Cary, NC, USA). Statistical significance was set at P < 0.05.

## Results

Table 1 shows the baseline characteristics of 25,029 participants aged 40–74 years (10,134 men and 14,895 women) according to the absence or presence of incident disabling dementia during follow-up. In both men and women, compared with the participants without disabling dementia, those with disabling dementia were more likely to be older and high-risk individuals who suffered from hypertension. In men, those with disabling dementia had a higher rate of smoking and lower means of LDL-cholesterol level and BMI, whereas women with disabling dementia had a lower rate of smoking and a higher mean LDL-cholesterol level. In women, those with disabling dementia had more diabetes mellitus and a lower mean HDL-cholesterol level.

**Table 1:** Age-adjusted baseline characteristics of the participants according to incident disabling dementia during follow-up.

	**Men** **(n = 10134)**			**Women** **(n = 14895)**		
	
	**Incident disabling dementia**		**P value for difference^a^**	**Incident disabling dementia**		**P value for difference^a^**
	
	**No**	**Yes**		**No**	**Yes**	
No. (%)	9528 (94.0)	606 (6.0)		14179 (95.2)	716 (4.8)	
Age, years	62.2 (9.5)	69.3 (4.5)	<0.0001	62.5 (8.6)	69.6 (4.0)	<0.0001
Current smoking, %	33.7	36.6	<0.0001	8.9	7.5	0.006
Current drinking, %	69.5	66.2	0.06	35.3	26.7	0.09
Body mass index, kg/m^2^	23.6 (3.1)	22.9 (3.2)	0.0007	22.6 (3.3)	22.6 (3.6)	0.05
Overweight (≥25 kg/m^2^)	29.3	24.3	0.32	20.7	21.2	0.30
Obesity (≥30 kg/m^2^)	2.9	2.0	0.78	3.1	3.8	0.28
Systolic blood pressure, mmHg	130.8 (18.6)	136.4 (19.0)	0.007	127.5 (18.9)	133.1 (18.4)	0.33
Diastolic blood pressure, mmHg	77.9 (11.5)	78.5 (11.7)	0.50	74.5 (11.0)	75.7 (10.9)	0.79
Use of antihypertensive medication, %	25.2	36.0	0.07	23.3	35.5	0.009
Hypertension, %	47.3	61.1	0.03	40.4	56.3	0.01
Fasting blood glucose, mmol/L	5.7 (1.5)	5.8 (1.8)	0.51	5.3 (1.0)	5.5 (1.2)	0.0007
HbA1c, %	5.7 (0.90)	5.8 (0.99)	0.54	5.6 (0.66)	5.8 (0.86)	0.25
Use of glucose-lowering medication, %	6.5	9.7	0.12	3.5	8.8	<0.0001
Diabetes mellitus, %	15.0	18.3	0.62	7.6	14.1	<0.0001
Serum triglycerides, mmol/L	1.6 (1.2)	1.5 (1.0)	0.45	1.2 (0.7)	1.3 (0.7)	0.98
Serum LDL-cholesterol, mmol/L	3.3 (0.8)	3.0 (0.8)	<0.0001	3.46 (0.84)	3.47 (0.84)	0.002
Serum HDL-cholesterol, mmol/L	1.5 (0.4)	1.5 (0.4)	0.86	1.72 (0.41)	1.71 (0.44)	0.046
Use of lipid-lowering medication, %	9.1	12.5	0.31	18.1	24.4	0.71

Values are reported as mean (standard deviation) or percentage.

^a^Adjusted for age.

During 211,804 person-years of follow-up (median follow-up of 9.1 years), 1,322 (606 men and 716 women) disabling dementia events occurred. The incidence of disabling dementia tended to be lower in women than in men, but the multivariable HR (95% CI) for women versus men was 0.89 (0.78–1.02).

Table 2 shows the sex-specific HRs and PAFs of incident disabling dementia according to the absence or presence of cardiovascular risk factors. Current smoking and hypertension were positively and similarly associated with a higher risk of disabling dementia in both sexes (p for sex interaction = 0.94 and 0.88, respectively), while overweight and obesity were not associated with the risk in either sex (p for sex interaction = 0.71 and 0.68, respectively). Diabetes mellitus was associated only in women (p for sex interaction = 0.04). In men, the PAF of hypertension was the highest, and that of smoking was the second-highest; the corresponding PAFs were 14.1 (95% CI, 4.8 to 22.4) and 13.4 (95% CI, 8.0 to 18.6), respectively. In women, the PAFs were high in the order of hypertension, diabetes mellitus, and smoking; the corresponding PAFs were 11.8 (4.0 to 18.9), 5.3 (2.4 to 8.1), and 3.1 (1.1 to 5.2), respectively. The total PAF of the significant risk factors was 27.5% in men and 20.2% in women.

**Table 2:** Sex-specific HRs and PAFs of incident disabling dementia according to cardiovascular risk factors.

	**Men** **(n = 10134)**						**Women** **(n = 14895)**						**P for interaction**
	
	**No. of persons**	**No. of events**	**person-years**	**Age-adjusted ** **HR (95% CI)**	**Multivariable ** **HR (95% CI)^a^**	**PAF** **(95% CI), %**	**No. of persons**	**No. of events**	**person-years**	**Age-adjusted ** **HR (95% CI)**	**Multivariable ** **HR (95% CI)^a^**	**PAF** **(95% CI), %**	
Current smoking													
Yes	3432	222	27848	1.60 (1.35–1.89)	1.58 (1.34–1.87)	13.4 (8.0 to 18.6)	1316	54	10728	1.61 (1.22–2.12)	1.71 (1.29–2.27)	3.1 (1.1 to 5.2)	0.94
No	6702	384	56056	1.00	1.00		13579	662	117172	1.00	1.00		
Overweight (≥25 kg/m^2^)													
Yes	2941	147	24132	0.92 (0.76–1.10)	0.95 (0.78–1.15)	-	3081	152	26312	0.93 (0.78–1.12)	0.90 (0.75–1.08)	-	0.71
No	7193	459	59772	1.00	1.00		11814	564	101588	1.00	1.00		
Obesity (≥30 kg/m^2^)													
Yes	292	12	2360	1.02 (0.58–1.81)	1.01 (0.57–1.79)	-	460	27	3829	1.31 (0.89–1.93)	1.20 (0.81–1.77)	-	0.68
No	9842	594	81544	1.00	1.00		14435	689	124071	1.00	1.00		
Hypertension													
Yes	4876	370	39935	1.29 (1.09–1.52)	1.30 (1.10–1.54)	14.1 (4.8 to 22.4)	6134	403	52649	1.26 (1.09–1.46)	1.26 (1.09–1.47)	11.8 (4.0 to 18.9)	0.88
No	5258	236	43969	1.00	1.00		8761	313	75251	1.00	1.00		
Diabetes mellitus													
Yes	1536	111	12360	1.16 (0.95–1.43)	1.10 (0.89–1.36)	-	1182	101	9979	1.59 (1.29–1.96)	1.60 (1.29–1.99)	5.3 (2.4 to 8.1)	0.04
No	8598	495	71544	1.00	1.00		13713	615	117921	1.00	1.00		

HR, hazard ratio; CI, confidence interval; PAF, population-attributable fraction.

^a^Adjusted for age, smoking status, drinking status, overweight or obesity, hypertension, diabetes mellitus, Serum LDL-cholesterol, Serum HDL-cholesterol, and lipid-lowering medication use.

Table 3 shows the sex- and age-specific HRs and PAFs of incident disabling dementia according to the absence or presence of cardiovascular risk factors. Current smoking in late-life (65–74 years) was associated with a higher risk of disabling dementia in both sexes. In contrast, there was no significant association between current smoking and disabling dementia in midlife (40–64 years), although the interaction by age was not significant in either sex. The PAFs of late-life smoking in men and women were 13.4 (95% CI, 7.4 to 18.4) and 2.9 (95% CI, 0.8 to 4.9), respectively. Overweight and obesity were not associated with risk of disabling dementia, regardless of sex and age. Hypertension was associated with an increased risk of disabling dementia in middle-aged men and older women, but not in older men and middle-aged women (P for interaction = 0.0003 in men and 0.17 in women). The PAFs of hypertension were 36.8 (95% CI, 14.4 to 53.3) in middle-aged men and 12.0 (95% CI, 3.7 to 19.7) in older women. Diabetes mellitus was associated with a higher risk of disabling dementia in both middle-aged and older women and the association was stronger in older women than in middle-aged women (P for interaction = 0.009). The PAFs of diabetes mellitus were 10.9 (95% CI, 0.01 to 20.6) in middle-aged women and 4.7 (95% CI, 1.57 to 7.6) in older women.

**Table 3:** Sex- and age-specific HRs and PAFs of incident disabling dementia according to each risk factor.

	**Men** **(n = 10134)**					**Women** **(n = 14895)**				
	
	**No. of persons**	**No. of events**	**Person-years**	**Multivariable ** **HR (95% CI)^a^**	**PAF** **(95% CI), %**	**No. of persons**	**No. of events**	**Person-years**	**Multivariable ** **HR (95% CI)^a^**	**PAF** **(95% CI), %**
<65 years										
Current smoking										
Yes	1855	35	14930	1.45 (0.92–2.27)	-	910	9	7360	1.51 (0.73–3.09)	-
No	2651	44	21650	1.00		6235	60	52847	1.00	
≥65 years										
Current smoking										
Yes	1577	187	12919	1.61 (1.34–1.92)	13.4 (7.7 to 18.7)	406	45	3368	1.72 (1.26–2.33)	2.9 (0.8 to 4.9)
No	4051	340	34406	1.00		7344	602	64325	1.00	
	P for interaction by age group = 0.37					P for interaction by age group = 0.45				
<65 years										
Overweight^b^										
Yes	1488	18	11976	0.70 (0.41–1.21)	-	1341	14	11246	0.84 (0.45–1.57)	-
No	3018	61	24604	1.00		5804	55	48960	1.00	
≥65 years										
Overweight^b^										
Yes	1453	129	12156	0.98 (0.80–1.21)	-	1740	138	15066	0.90 (0.74–1.10)	-
No	4175	398	35168	1.00		6010	509	52628	1.00	
	P for interaction by age group = 0.13					P for interaction by age group = 0.63				
<65 years										
Obesity^c^										
Yes	188	2	1489	0.78 (0.19–3.25)	-	218	5	1777	2.03 (0.78–5.33)	-
No	4318	77	35091	1.00		6927	64	58430	1.00	
≥65 years										
Obesity^c^										
Yes	104	10	870	1.03 (0.55–1.93)	-	242	22	2053	1.09 (0.71–1.69)	-
No	5524	517	46454	1.00		7508	625	65641	1.00	
	P for interaction by age group = 0.48					P for interaction by age group = 0.13				
<65 years										
Hypertension										
Yes	1706	51	13724	2.32 (1.44–3.76)	36.8 (14.4 to 53.3)	2191	32	18660	1.21 (0.74–1.99)	-
No	2800	28	22856	1.00		4954	37	41546	1.00	
≥65 years										
Hypertension										
Yes	3170	319	26211	1.19 (0.995–1.42)	-	3943	371	33989	1.27 (1.08–1.48)	12.0 (3.7 to 19.7)
No	2458	208	21113	1.00		3807	276	33704	1.00	
	P for interaction by age group = 0.0003					P for interaction by age group = 0.17				
<65 years										
Diabetes mellitus										
Yes	568	14	4486	1.09 (0.60–1.98)	-	413	13	3497	2.38 (1.25–4.55)	10.9 (0.01 to 20.6)
No	3938	65	32094	1.00		6732	56	56709	1.00	
≥65 years										
Diabetes mellitus										
Yes	968	97	7873	1.09 (0.87–1.36)	-	769	88	6482	1.53 (1.21–1.93)	4.7 (1.7 to 7.6)
No	4660	430	39451	1.00		6981	559	61212	1.00	
	P for interaction by age group = 0.41					P for interaction by age group = 0.009				

HR, hazard ratio; CI, confidence interval; PAF, population-attributable fraction.

^a^Adjusted for age, smoking status, drinking status, overweight or obesity, hypertension, diabetes mellitus, Serum LDL-cholesterol, Serum HDL-cholesterol, and lipid-lowering medication use.

^b^BMI ≥25 kg/m^2^

^c^BMI ≥30 kg/m^2^

## Discussion

This community-based prospective study of over 25,000 dementia-free Japanese individuals showed that smoking and hypertension were associated with an increased risk of disabling dementia in both sexes and diabetes mellitus in women. Moreover, we found that hypertension was associated with the risk in midlife than in late-life among men, and diabetes mellitus was stronger associated with the risk in late-life than in midlife among women. Our estimate of PAF indicated that 28% of male disabling dementia cases and 20% of female disabling dementia cases could be prevented by modifying these risk factors. To the best of our knowledge, our study is the first to evaluate the sex- and age-specific impacts of these risk factors on the risk of disabling dementia.

The smoking rate has been high in Japanese men, although the rate declined over the years from 24.2% in 2009 to 17.7% in 2019, while in contrast, the rate has been low in women from 11.7% in 2009 to 8.1% in 2019 [30]. The larger burden of dementia attributable to smoking in men than in women in the present study was due to men’s higher smoking rate despite the similar association between smoking and dementia in both sexes.

In the present study, overweight or obesity was not associated with the risk of disabling dementia, regardless of sex or age, probably because of the low prevalence of overweight (24%) and obesity (3%) compared with non-Asian populations. Our result was consistent with the finding from a previous study in older Japanese [9].

Hypertension has been acknowledged to be a strong risk factor for mortality and morbidity from stroke among Japanese [17], and midlife high blood pressure has been reported as a significant predictor of reduced cognitive function and increased dementia incidence in later life in the Honolulu-Asia Aging Study [31, 32], the Hisayama study [33], and a Chinese study [34]. In the present study, midlife hypertension in men and late-life hypertension in women associated with the risk of disabling dementia.

We found that diabetes mellitus was a strong predictor for disabling dementia in women but not in men, which was consistent with the finding from a pooled analysis of 2.3 million people comprising more than 100,000 cases of dementia in 11 Western and 3 Asian studies [15]; women with diabetes had a 19% greater risk of vascular dementia than men (the women-to-men ratio of the pooled multiple-adjusted relative risks 1.19 [95% CI 1.08–1.30]; p < 0.001), but not for non-vascular dementia. Diabetic women had a larger number of other cardiovascular risk factors than diabetic men, which may be due to the greater increase in adiposity and insulin resistance associated with diabetes mellitus in women than in men [35].

Potential mechanisms for the development of dementia by smoking, hypertension, and diabetes mellitus are as follows. Smoking and diabetes mellitus increase oxidative stress which may enhance the development of arteriolosclerosis in cerebral arteries (small vessel disorders), leading to vascular dementia [36, 37], and also may initiate and progress the accumulation of amyloid and tau proteins, leading to an increased risk of Alzheimer’s disease [38]. Hypertension is a strong determinant of arteriolosclerosis in cerebral arteries, leading to vascular dementia [39]. There is no good explanation on the more evident contribution of diabetes mellitus to dementia in women than in men. A recent cross-sectional imaging study of older persons showed that diabetes mellitus was associated with cerebral lacunes, brain atrophy, and impaired cognitive function in women but not in men, possibly due to more oxidative stress and pro-thrombotic state in women [40].

The strengths of our study include a large population-based prospective study of over 25,000 dementia-free individuals and the first study to systematically evaluate the sex-specific impacts of cardiovascular risk factors on the risk of disabling dementia. Our finding from the large population-based sample could be extrapolated to general Japanese populations. Moreover, we evaluated the impact of midlife risk factors for dementia, although a previous study of 8,563 older Japanese individuals aged 65 years or older reported those of late-life risk factors [9].

Our study has several limitations. First, because a detailed clinical diagnosis of dementia was not obtained, we did not evaluate the types of dementia, such as Alzheimer’s disease or vascular dementia. Second, some misclassification of diagnosis for incident dementia could occur in the present study. Because our dementia criteria had sensitivity (73%) and specificity (96%) against clinical diagnosis by neuropsychiatrists [22], which unlikely biased the estimation of the association mathematically. Third, we did not examine the potential risk factors such as less education, hearing loss, traumatic brain injury, depression, social isolation, physical inactivity, and air pollution due to no data availability. Further studies are needed to build evidence on the associations between modifiable risk factors and the risk of dementia, especially in Asian populations.

## Conclusion

In conclusion, a substantial burden of disabling dementia was attributable to smoking and hypertension in both sexes and diabetes mellitus in women, which may require the management of smoking, hypertension, and diabetes mellitus to prevent dementia.

## Abbreviations

BMI: Body mass index
SBP: Systolic blood pressure
DBP: Diastolic blood pressure
LDL-C: Low-density lipoprotein cholesterol
HDL-C: High-density lipoprotein cholesterol
HbA1c: Haemoglobin A1c
JDS: Japan Diabetes Society
NGSP: National Glycohemoglobin Standardization Program
HR: Hazard ratio
CI: Confidence interval
PAF: Population attributable fraction

## Declarations

### Availability of data and material

Data cannot be shared for ethical reasons: informed consent has not been obtained from the participants for the open use of their personal data.
